# Vaccine-associated measles in a patient treated with natalizumab: a case report

**DOI:** 10.1186/s12879-020-05475-9

**Published:** 2020-10-14

**Authors:** Alix Miauton, Rainer Tan, Vasiliki Pantazou, Renaud Du Pasquier, Blaise Genton

**Affiliations:** 1 Tropical, travel and vaccination clinic, Unisanté, Center for primary care and public health, Bugnon 44, 1011 Lausanne, Switzerland; 2 Department of Neurology, Lausanne University Hospital, Bugnon 46, 1011 Lausanne, Switzerland

**Keywords:** Case report, Measles mumps rubella vaccine, Measles, Natalizumab, Immunosuppression, Vaccine adverse event, Multiple sclerosis, Vaccine

## Abstract

**Background:**

Safety of live vaccines in patients treated with immunosuppressive therapies is not well known, resulting in contradictory vaccination recommendations. We describe here the first case of vaccine-associated measles in a patient on natalizumab treatment.

**Case presentation:**

A young female patient with relapsing-remitting multiple sclerosis on natalizumab treatment received the live attenuated measles, mumps, and rubella vaccine in preparation for a change in her treatment in favour of fingolimod, with established immunosuppressive qualities. Seven days after receiving the vaccine, our patient experienced diffuse muscle pain, fatigue, and thereafter developed a fever and then an erythematous maculopapular rash, compatible with vaccine associated measles. This was later confirmed by a positive measles RT-PCR throat swab. The patient’s symptoms resolved without any sequelae.

**Conclusion:**

In this case report we review the immunosuppressive qualities of natalizumab and the evidence in favour and against live vaccines in patients on this treatment. Our findings reveal the insufficient understanding of the immunosuppressive effects of new immunomodulators, and thus of the safety of live vaccines in patients on such medications. While this case triggers precaution, there is insufficient evidence to conclude that natalizumab treatment could favor the onset of vaccine-associated measles.

## Background

Live-attenuated vaccines, including the Measles-Mumps-Rubella (MMR) vaccine, consist of an attenuated microorganism still capable of replicating. They are generally contraindicated in patients with immunosuppressive therapies, by fear of systemic infection due to vaccine strain replication [1]. Paradoxically, those patients are more vulnerable to infectious diseases and would particularly benefit from immunization. Emergence of increasingly diverse immunosuppressive treatments, whose immunologic effect is not always clear, challenges this general contraindication. At present, little data exists on the safety of live-attenuated vaccines in those patients, and recommendations are based on expert opinion that may differ [2–6].

We describe here the first known case of vaccine-associated measles in a young female vaccine recipient with relapsing-remitting multiple sclerosis (RRMS), treated with natalizumab. Our aim is to explore the safety of vaccination in patients with multiple sclerosis (MS), the immunosuppressive qualities of natalizumab and the evidence in favour and against live vaccines in patients on this treatment.

## Case presentation

A 35-year-old patient with RRMS treated with natalizumab injections for the past 3 years wanted to switch to an oral medication for practical reasons. She was therefore referred to our vaccination clinic in order to update her vaccinations before switching to fingolimod, an oral RRMS treatment with established immunosuppressive properties. Given a lack of immunity to measles (IgG negative), she was given the MMR vaccine following the local recommendations that do not contraindicate live attenuated vaccines in patients treated with natalizumab [2]. She was also immunized with the hepatitis A, influenza, diphtheria, tetanus, pertussis and pneumococcal 13-valent vaccines. Seven days post vaccination, she started to experience diffuse muscle pain and fatigue. At day 9 post vaccination, she developed a fever (38.5–39 °C), and 2 days later she complained of a sore throat and an erythematous maculopapular rash that spread initially from the trunk and face, to the extremities. She did not experience coryza, conjunctivitis or respiratory symptoms. No travel history was reported, nor infectious exposures. At day six of symptoms, she consulted a general medicine outpatient clinic. The physical exam revealed an erythematous maculopapular rash, posterior right cervical and submandibular lymph nodes, and slightly inflamed tonsils. No strawberry tongue or Koplik spots were noted. Blood exams found a slightly elevated C-reactive protein at 47 mg/dL, and normal white blood cell count (6G/L). A rapid streptococcus throat test was positive, which led the clinician to suspect either acute streptococcal pharyngitis with scarlet fever or vaccine-associated measles. Amoxicillin 500 mg twice daily was prescribed for six days. Two days after the start of antibiotics, her fever broke and she described an improvement in all of her symptoms. Eight days after the start of the antibiotics, when she sought medical attention in our vaccination clinic, she was asymptomatic, and her physical examination was unremarkable other than her fading rash without any desquamation. A measles RT-PCR throat swab was performed at that time and the result came back positive at 1100 copies/ml. Other common respiratory viruses (influenza, respiratory syncytial virus) were negative. Typisation of the measles strain was not possible due to the small quantity of RNA.

Without the typisation of the measles strain, we cannot ascertain that this was truly a case of vaccine-associated measles. Indeed, wild type measles could not be excluded, however the patient did not report any contact with cases of measles or any sick contact. The differential diagnosis of scarlet fever, another viral febrile rash, or simply vaccine side-effects was also considered. Nevertheless, the timeline of the vaccination and symptoms correspond typically with a case of vaccine-associated measles. Given this high probability, no formal contact tracing was conducted by Public Health authorities and no secondary cases were reported.

Upon communicating the results of the measles throat swab and our diagnostic assessment, the patient was initially concerned about long lasting repercussions despite being asymptomatic. Following a brief discussion, these concerns were allayed.

## Discussion and conclusions

This is the first case of a vaccine-associated measles in a patient treated with natalizumab, based on a structured search of PubMed, the Vaccine Adverse Event Reporting System and VigiBase, the WHO global database of individual case safety reports (ICSRs).

Measles vaccination has come back in the spotlight in Europe and in the United States with a sharp resurgence of measles cases since 2018, including in countries that had previously interrupted endemic transmission [7, 8]. The measles vaccine is very effective, with on average 95.7% protection following the first vaccination [9]. While mild side effects are common (fever, transient rash and transient lymphadenopathy in about 15, 5 and 20% of recipients, respectively), serious adverse effects are rare (febrile seizures − 1 case per 3′000 doses-, thrombocytopenic purpura − 1 case per 40′000 doses-, and anaphylaxis – 1.8 to 14.4 cases per million doses) [10]. Only a few cases of vaccine-associated measles have been reported, both in immunocompetent [11, 12] and immunocompromised [13–15] patients (real number of cases probably higher due to underreporting). Altogether, these risks are clearly inferior to those observed with a natural measles infection [16].

While benefit of measles vaccination appears indisputable in healthy individuals, several aspects challenge the risk-benefit ratio of measles immunization in the patient described above, with MS treated with natalizumab.

Prevention of infection by vaccination is indeed an integral part of the management of patients with MS. [17] Systemic infections have been shown to worsen clinical symptoms of MS (pseudo-exacerbations) and may increase the risk of MS relapse [18]. While there were once concerns about vaccine safety in patients with MS, two systematic reviews found no evidence of an association between the most common vaccinations (including the MMR vaccine) and the onset of MS. [19, 20] Furthermore, no association was found between the risk of MS relapse and influenza, H1N1, Hepatitis B and tetanus vaccines. However, evidence is lacking in regards to the MMR vaccine [19].

The effect of natalizumab on vaccine tolerance may also be questioned. Natalizumab is an immunosuppressive treatment approved since 2004 for RRMS and administered monthly by intravenous infusion. Over 200'000 people have been treated with natalizumab worldwide [21]. It is a recombinant humanized monoclonal antibody that inhibits the α4-integrin subunit on leukocytes that has shown great clinical efficacy [22–24]. By blocking these cell-surface receptors, it reduces leukocytes ability to adhere on brain endothelium and thus to cross the blood-brain barrier [25]. Patients treated with natalizumab present an inverted CD4+/CD8+ ratio in cerebrospinal fluid, a finding often associated with central nervous system (CNS) immunosuppression and opportunistic infections [26]. Indeed, natalizumab has been associated with a higher risk of progressive multifocal leukoencephalopathy, a CNS opportunistic infection caused by reactivation of a latent human polyomavirus 2 (formerly JC virus) [27], and possibly CNS herpesvirus infection [28]. While CNS immunosuppression by natalizumab treatment is well established, the extent of its non-CNS immunosuppression remains to be demonstrated. In fact, even if a decrease of IgG and IgM levels in the blood of patients on natalizumab treatment has been reported [29], clinical studies have not found significantly higher incidences of non-CNS infections compared to placebo groups and general population cohorts [22, 30].

Nevertheless, while safety of inactivated vaccines (influenza, hepatitis B, human papillomavirus, tetanus vaccines) has been well established in patients treated with natalizumab [31], data is scarce when it comes to live attenuated vaccine tolerance, such as the MMR vaccine. This has led international guidelines to advise against the administration of the MMR vaccine in all patients treated with immunosuppressive therapies including the new biotherapies for MS due to fears of vaccine-associated disease [5, 6, 31]. More specifically, French [4] and Canadian [3] recommendations argue that despite no supporting data, patients treated by natalizumab should be considered as immunocompromised and thus not be vaccinated with MMR. Yet a retrospective study of 116 patients on immunosuppressive therapy (including one patient on natalizumab treatment) matched with a healthy control group found no difference in local and systemic reactions after receiving a live attenuated vaccine [32]. Moreover, several other studies did not find any worrying adverse effects after MMR vaccination in patients with autoimmune diseases treated by methotrexate and/or biological agents [33] nor in patients on immunosuppressive therapy after solid organ transplantation [34]. These results and the lack of evidence of a non-CNS immunosuppressive effect of natalizumab have led Swiss experts to state that live attenuated vaccines could be given to patients under such treatment after careful risk/benefit ratio assessment [2].

There is insufficient evidence to conclude that natalizumab treatment could favor the onset of vaccine-associated measles, as this case is isolated and vaccine- associated measles have also been described in immunocompetent patients. Nevertheless, this case calls for caution when using live attenuated vaccines in patients treated with immunosuppressive drugs, even without theoretical or proven systemic immunosuppressive effect. This must of course be balanced with the risk of acquiring wild-type measles, all the more in these vulnerable patients. Finally, this case demonstrates the importance of updating vaccinations before starting any immunosuppressive therapy, and strengthening population wide vaccination coverage to improve herd protection for the increasing number of patients on immunomodulatory and immunosuppressive drugs.

## Data Availability

Data sharing is not applicable to this article as no datasets were generated or analysed during the current study.

## Abbreviations

°C: Degree Celsius
CD4+: T-lymphocyte cell bearing CD4 receptor
CD8+: T-lymphocyte cell bearing CD8 receptor
CNS: Central Nervous System
dL: Deciliter
RNA: Ribonucleic acid
G/L: Giga per litre
ICRS: Individual case safety reports
IgG: Immunoglobulin G
IgM: Immunoglobulin M
JC: John Cunningham
mg: Milligram
MMR: Mumps, Measles, and Rubella
MS: Multiple Sclerosis
RT-PCR: Reverse transcription polymerase chain reaction
RRMS: Relapsing-remitting multiple sclerosis

## References

[1] Rubin LG, Levin MJ, Ljungman P, Davies EG, Avery R, Tomblyn M (2014). 2013 IDSA clinical practice guideline for vaccination of the immunocompromised host. Clin Infect Dis.

[2] Eperon G, Buhler S, Enriquez N, Vaudaux B (2018). The immunosuppressed traveler: vaccination guidelines. Rev Med Suisse.

[3] O'Connor PW, Kremenchutzky M (2015). Use of Natalizumab in patients with multiple sclerosis: 2015 update. Can J Neurol Sci Le J Canadien des Sciences Neurologiques.

[4] Lebrun C, Vukusic S, French Group for Recommendations in Multiple Sclerosis (2019). Immunization and multiple sclerosis: recommendations from the French multiple sclerosis society. Mult Scler Relat Disord.

[5] Loebermann M, Winkelmann A, Hartung HP, Hengel H, Reisinger EC, Zettl UK (2012). Vaccination against infection in patients with multiple sclerosis. Nat Rev Neurol.

[6] Epstein DJ, Dunn J, Deresinski S (2018). Infectious complications of multiple sclerosis therapies: implications for screening, prophylaxis, and management. Open Forum Infect Dis.

[7] The Centralized information system for infectious diseases (CISID). http://data.euro.who.int/cisid/. Accessed 26 May 2020.

[8] Centers for Disease Control and Prevention Newsroom Releases. https://www.cdc.gov/media/releases/2019/p0530-us-measles-2019.html. Accessed 26 May 2020.

[9] Organisation WH. The immunological Basis for Immunization Series. Module 7: Measles. Update 2009.

[10] McLean HQ, Fiebelkorn AP, Temte JL, Wallace GS, Centers for Disease Control and Prevention (2013). Prevention of measles, rubella, congenital rubella syndrome, and mumps, 2013: summary recommendations of the advisory committee on immunization practices (ACIP). MMWR Recomm Rep.

[11] Sood SB, Suthar K, Martin K, Mather K (2017). Vaccine-associated measles in an immunocompetent child. Clin Case Rep.

[12] Kurata T, Kanbayashi D, Kinoshita H, Arai S, Matsui Y, Fukumura K (2014). Late onset of vaccine-associated measles in an adult with severe clinical symptoms: a case report. Am J Med.

[13] Angel JB, Walpita P, Lerch RA, Sidhu MS, Masurekar M, DeLellis RA (1998). Vaccine-associated measles pneumonitis in an adult with AIDS. Ann Intern Med.

[14] Monafo WJ, Haslam DB, Roberts RL, Zaki SR, Bellini WJ, Coffin CM (1994). Disseminated measles infection after vaccination in a child with a congenital immunodeficiency. J Pediatr.

[15] Bitnun A, Shannon P, Durward A, Rota PA, Bellini WJ, Graham C (1999). Measles inclusion-body encephalitis caused by the vaccine strain of measles virus. Clin Infect Dis.

[16] Strebel PM, Orenstein WA (2019). Measles. N Engl J Med.

[17] Rutschmann OT, McCrory DC, Matchar DB, Immunization Panel of the Multiple Sclerosis Council for Clinical Practice G (2002). Immunization and MS: a summary of published evidence and recommendations. Neurology..

[18] Williamson EM, Berger JR (2015). Infection risk in patients on multiple sclerosis therapeutics. CNS Drugs.

[19] Mailand MT, Frederiksen JL (2017). Vaccines and multiple sclerosis: a systematic review. J Neurol.

[20] Stratton K, Ford A, Rusch E, Clayton EW, Medicine Io (2012). Adverse effects of vaccines: evidence and causality.

[21] Biogen (2019). New research demonstrate Biogen's continued commitment to improve care of patients with multiple sclerosis across treatment spectrum.

[22] Polman CH, O'Connor PW, Havrdova E, Hutchinson M, Kappos L, Miller DH (2006). A randomized, placebo-controlled trial of natalizumab for relapsing multiple sclerosis. N Engl J Med.

[23] Kalincik T, Brown JWL, Robertson N, Willis M, Scolding N, Rice CM (2017). Treatment effectiveness of alemtuzumab compared with natalizumab, fingolimod, and interferon beta in relapsing-remitting multiple sclerosis: a cohort study. Lancet Neurol.

[24] Rudick RA, Stuart WH, Calabresi PA, Confavreux C, Galetta SL, Radue E-W (2006). Natalizumab plus interferon Beta-1a for relapsing multiple sclerosis. N Engl J Med.

[25] Noseworthy JH, Kirkpatrick P (2005). Natalizumab. Nat Rev Drug Discov.

[26] Stuve O, Marra CM, Bar-Or A, Niino M, Cravens PD, Cepok S (2006). Altered CD4+/CD8+ T-cell ratios in cerebrospinal fluid of natalizumab-treated patients with multiple sclerosis. Arch Neurol.

[27] Bloomgren G, Richman S, Hotermans C, Subramanyam M, Goelz S, Natarajan A (2012). Risk of natalizumab-associated progressive multifocal leukoencephalopathy. N Engl J Med.

[28] Yao K, Gagnon S, Akhyani N, Williams E, Fotheringham J, Frohman E (2008). Reactivation of human herpesvirus-6 in natalizumab treated multiple sclerosis patients. PLoS One.

[29] Warnke C, Stettner M, Lehmensiek V, Dehmel T, Mausberg AK, von Geldern G (2015). Natalizumab exerts a suppressive effect on surrogates of B cell function in blood and CSF. Mult Scler.

[30] Luna G, Alping P, Burman J, Fink K, Fogdell-Hahn A, Gunnarsson M (2019). Infection risks among patients with multiple sclerosis treated with Fingolimod, Natalizumab, rituximab, and injectable therapies. JAMA Neurol.

[31] Williamson EM, Chahin S, Berger JR (2016). Vaccines in multiple sclerosis. Curr Neurol Neurosci Rep.

[32] Huber F, Ehrensperger B, Hatz C, Chappuis F, Buhler S, Eperon G. Safety of live vaccines on immunosuppressive or immunomodulatory therapy-a retrospective study in three Swiss travel clinics. J Travel Med. 2018;25(1). 10.1093/jtm/tax082 29394383

[33] Heijstek MW, Kamphuis S, Armbrust W, Swart J, Gorter S, de Vries LD (2013). Effects of the live attenuated measles-mumps-rubella booster vaccination on disease activity in patients with juvenile idiopathic arthritis: a randomized trial. JAMA..

[34] Shinjoh M, Hoshino K, Takahashi T, Nakayama T (2015). Updated data on effective and safe immunizations with live-attenuated vaccines for children after living donor liver transplantation. Vaccine..

